# Colorectal cancer patients-derived immunity-organoid platform unveils cancer-specific tissue markers associated with immunotherapy resistance

**DOI:** 10.1038/s41419-024-07266-5

**Published:** 2024-12-04

**Authors:** A. Esposito, A. Agostini, G. Quero, G. Piro, L. Priori, A. Caggiano, G. Scaglione, A. Battaglia, M. A. Calegari, L. Salvatore, M. Bensi, M. G. Maratta, A. Ceccarelli, G. Trovato, G. Genovese, E. Gurreri, S. Ascrizzi, M. Martini, C. Fiorillo, A. Fattorossi, F. De Sanctis, S. Ugel, V. Corbo, S. Alfieri, G. Tortora, C. Carbone

**Affiliations:** 1 Medical Oncology, Department of Translational Medicine, Catholic University of the Sacred Heart, Rome, Italy; 2 Medical Oncology, Department of Medical and Surgical Sciences, Fondazione Policlinico Universitario A. Gemelli IRCCS, Roma, Italy; 3 Pancreatic Surgery Unit, Gemelli Pancreatic Advanced Research Center (CRMPG), Fondazione Policlinico Universitario A. Gemelli IRCCS, Rome, Italy; 4 Digestive Surgery Unit, Department of Translational Medicine, Catholic University of the Sacred Heart, Rome, Italy; 5 Department of Anatomic Pathology, Fondazione Policlinico Universitario A. Gemelli IRCCS, Rome, Italy; 6 Department of Life Science and Public Health, Catholic University of the Sacred Heart, Rome, Italy; 7 Department of Genitourinary Medical Oncology, Division of Cancer Medicine, The University of Texas MD Anderson Cancer Center, Houston, TX USA; 8 Department of Human Pathology in Adult and Developmental Age “Gaetano Barresi”, Pathology Section, University of Messina, Messina, Italy; 9 Section of Immunology, Department of Medicine, University of Verona, Verona, Italy; 10 Department of Diagnostics and Public Health, University of Verona, Verona, Italy

**Keywords:** Cancer, Biomarkers

## Abstract

Colorectal cancer (CRC) is a devastating disease, ranking as the second leading cause of cancer-related deaths worldwide. Immune checkpoint inhibitors (ICIs) have emerged as promising treatments; however, their efficacy is largely restricted to a subgroup of microsatellite instable (MSI) CRCs. In contrast, microsatellite stable (MSS) CRCs, which account for the majority of cases, exhibit variable and generally weaker response to ICIs, with only a subset demonstrating exceptional responsiveness. Identifying novel cancer-specific tissue (CST) markers predictive of immunotherapy response is crucial for refining patient selection and overcoming treatment resistance. In this study, we developed clinically relevant CRC organoids and autologous immune system interaction platforms to model ICI response. We conducted a comprehensive molecular characterization of both responder and non-responder models, identifying CST markers that predict ICI response. Validation of these findings was performed using an independent cohort of patient specimens through multiplex immunofluorescence. Furthermore, we demonstrated that knocking out a key gene from the identified predictive signature in resistant organoids restored immune sensitivity and induced T-cell-mediated apoptosis. Overall, our results provide novel insights into the mechanisms underlying immunotherapy resistance and suggest new markers for enhancing patient selection. These findings may pave the way for new therapeutic options in MSS patients, potentially broadening the cohort of individuals eligible for immunotherapy.

## Introduction

Colorectal Cancer (CRC) is the third most common malignancy and the second leading cause of cancer-related deaths worldwide, with a 5-year survival rate of approximately 65% [1, 2]. CRC is typically categorized based on microsatellite stability into two main groups: Microsatellite Stable (MSS) tumors and Microsatellite Instable (MSI) tumors. This classification influences various aspects of the cancer, including its development, progression, treatment response, and prognosis [3, 4]. In MSI tumors, the defect of mismatch repair causes the accumulation of mutations in microsatellites and results in elevated mutational rate, along with increased expression of neo-antigens [3, 5]. These molecular features are responsible for recruiting immune cells, particularly tumor-infiltrating lymphocytes (TILs) [6]. Conversely, MSS tumors often develop an immunosuppressive tumor microenvironment (TME), characterized by the recruitment of Myeloid-Derived Suppressor Cells (MDSCs) [7, 8].

In recent years, immunotherapy revolutionized the treatment approach of solid tumors, including CRCs, offering new hope for patients [9, 10]. Indeed, immune checkpoint inhibitors (ICIs) have demonstrated remarkable clinical effectiveness in MSI CRCs (accounting for only 15% of all CRC cases), while their efficacy has generally been limited in MSS CRCs enrolled in the first monotherapy clinical trials [11, 12]. Notably, approximately 25–40% of MSI patients exhibit primary resistance to ICIs, while others may develop resistance during treatment [13–15]. The underlying mechanisms of these varied responses remain poorly understood, underscoring the need for deeper exploration. Moreover, some MSS patients have demonstrated significant responses to immunotherapy, further highlighting the importance of improved patient selection strategies [16, 17]. MSS CRCs have long been thought to be refractory to immunotherapy in unselected cohorts due to the lack of neoantigens surface expression. However, recent drug combination studies indicate that, in some cases, immunotherapy still has the potential to play an important role in the treatment of MSS CRC [18–21]. Interestingly, a recent comprehensive genomic analysis of a large series of CRCs has revealed substantial heterogeneity within the MSS group, demonstrating that MSS CRCs are not a homogenous entity and identifying four distinct subgroups with independent prognostic and molecular features, some of which resemble MSI tumors [22].

Patient-derived CRC organoids (PDOs) have been extensively utilized in preclinical research for their ability to accurately represent the genomic characteristics of tumors [23, 24]. However, PDOs alone do not fully capture the complexities of the TME, which plays a critical role in therapeutic responses [25, 26]. Co-culturing organoids with TME components can overcome this limitation offering a more physiologically relevant model for cancer research and therapy development [27–29].

Here we established ex vivo autologous Patient Derived Immuno-Organoids (PD-IOs) interaction platforms to recapitulate the complex interplay between tumor and key immune components, T-cells and MDSCs. Our aim was to identify potential cancer-specific tissue (CST) markers of resistance to immunotherapy. The identified markers were validated in a retrospective cohort of MSI CRC patients, in which immunotherapy achieved complete pathological response, partial response or no response. Our findings revealed new markers beyond microsatellite stability status that may enhance patient selection by identifying MSI patients who may not respond to ICIs, thereby preventing them from experiencing adverse events of an ineffective therapy. More importantly, they suggest new possibilities for treating a selected subgroup of MSS patients.

## Methods

### Patient Material

Material for the study was acquired from patients who received treatment at Fondazione Policlinico Universitario “Agostino Gemelli” between 2020 and 2022 for colon cancer. 129 patients were screened for microsatellite stability. Fresh surgically resected colon cancer samples from 10 patients (6 MSS and 4 MSI) were used to establish organoids. Two expert pathologists confirmed the macroscopic presence of cancer in each sample. Each tumor was portioned for organoids establishment and tissue preservation for subsequent analysis. For marker validation, we utilized a set of 9 FFPE samples obtained from MSI patients who underwent immunotherapy treatment at our institution between 2020 and 2022.

### CRC Patient Genomic Profiling

In order to evaluate microsatellite stability status, we extracted genomic DNA from a total of 129 FFPE samples utilizing the QIAamp DNA FFPE Tissue kit (56404, Qiagen). Subsequently, targeted DNA sequencing was carried out using the Illumina TruSight Oncology 500 (TSO500) on the Illumina NovaSeq 6000 platform. The raw DNA sequencing data was then analyzed with TruSight Oncology 500 local application version 2.2. To validate the matching between organoids and their cancer of origin we performed Whole Exome Sequencing (WES) on three PDOs and their matched frozen tissues. We extracted genomic DNA with Quick-DNA tissue kit (Zymo Research). We utilized xGen DNA EZ Library Prep to obtain WES libraries that were after sequenced with NovaSeq 600. WES raw data were aligned with nf-core pipeline sarek 3.1.2.

### Whole Exome Sequencing (WES) and Transcriptomic analysis

DNA was extracted from organoids and matched tissues and WES was performed. WES raw data was analyzed with GATK and vcf files were annotated with ANNOVAR. RNA was extracted from organoids and tissues and sequenced for 3’UTR RNA-seq with Lexogen QUANTSEQ 3’ RNA FWD kit. RNA-seq raw data were aligned with STAR and differential expression analysis (DEA) was performed with DEseq2 R library. Differential expressed genes (Log2 Fold Change > 1.5 and adjusted *p*-value < 0.05) were used for gene set enrichment analysis (GSEA) with the clusteRprofiler package interrogating the msigDB gene signature database. TCGA data was obtained with TCGABiolinks R package. Immunodeconvolution on both CRC tissues and TCGA data was performed using the immunodeconv R package using the MCP-counter algorithm.

### Establishment of organoid cultures

Patient derived organoids (PDOs) were established from surgical resection from CRC patient of the Digestive Surgery of Policlinico Universitario Agostino Gemelli IRCCS, according to the protocol of Sato et al. [24]. Briefly, tumor tissue was washed with 100ug/ml primocin (ant-pm-2, InvivoGen) enriched-PBS, minced and then incubated in Gentle Cell Digestion Medium (GCDR (#100-0485, StemCell Technologies)) at 37° for 1 h. Crypts were removed from the tissue by vigorous pipetting. Digestion was blocked with Washing Medium (DMEM F12 Advanced - 12634-010, Gibco, 15 mM Hepes - 15630-080, Gibco, 1% BSA - A1391, Applichem) then filtered through a 70 μm strainer. Tumoral crypts were centrifuged at 290rcf for 6 min at RT, washed twice with Washing Medium and finally plated in 50 μl/dome in Cultrex UltiMatrix Reduced Growth Factor Basement Membrane Extract (BME001-10, R&D systems), at the concentration of 5000 crypts/well. After the incubation at 37 °C for 10 min to allow domes to solidify, 500 μl/well of pre-warmed tumor organoids medium IntestiCult™ Organoid Growth Medium (#06010, StemCell Technologies) were added.

### REG4 CRISPR-Cas9 mediated knockout

For PDOs REG4 knockout experiments sgRNA A (TTACGGAAACGGAGCCCACC) and sgRNA_B (GACTTGTGGTAAAACCATCC) were selected from IDT’s library predesigned guides. Both guides were used to determine which one resulted in the best knockout efficiency. Briefly, sgRNAs and Cas9-GFP (Alt-R™ S.p. Cas9-GFP V3) (IDT, Integrated DNA Technologies, Inc., Coralville, Iowa, USA) were mixed in equimolar ratio to form RNP complexes at RT for 20 min in the dark. PDOs were harvested and dissociated into single cells by digestion with TrypLE Express Enzyme (12605028, Gibco) for 15 min while shaking. To remove any residue of media-derived RNAses, PDOs were washed two times in PBS and counted to have 1 × 10^6^ cells/guide. For each condition/guide, PDOs were resuspended in 100 μl Opti-MEM (31985070, Gibco, Thermo Fisher Scientific Inc., Waltham, MA USA) containing respective RNP complex and Electroporation Enhancer (Alt-R® Cas9 Electroporation Enhancer, IDT). Electroporation was performed with NEPA21 gene electroporator. Electrical impedance was considered acceptable if within the 30–50 mΩ range. Electroporated PDOs mixture was recovered with pre-warmed tumor organoids medium and successively plated in 30 μl/dome in Cultrex UltiMatrix. Media were changed 24 h after electroporation and RNA was isolated after 72 h. GFP-positive cas9 was used to monitor RNP complexes internalization and efficiency was determined by Real-Time PCR (RT-PCR) and immunohistochemistry (IHC).

### Immunohistochemistry (IHC) and Immunofluorescence (IF) on PDOs and tumor tissues

Stabilized PDOs were resuspended in Histogel (HG-4000-012, Thermo Fisher Scientific) to perform FFPE inclusion. Briefly, tumor organoids cultured in Cultrex UltiMatrix domes were incubated in 2% Paraformaldehyde (PFA) for 5 min at 4 °C. Then, tumor organoids were collected with PBS by using a blunt micro-pipette tip and incubated in ice for 45 min. After a brief centrifugation, PFA and residual Cultrex were discarded, and 3D cultures were included in a mold with Histogel and left on ice for 3 min. The included 3D cultures were then moved in a histology cassette and incubated in 2% PFA overnight at 4 °C. The next day 3D cultures were included in Paraffin.

Whole 5 µm tissue sections from both PDOs and tumor tissues were dewaxed and rehydrated. Hematoxylin and Eosin stains were performed according to standard protocols. For IHC Antigen retrieval was performed using Bond Epitope Retrieval Solution 2 (AR9640, Leica Microsystems). The following antibodies were used for PDOs and primary tumor tissues immunohistochemical staining: CDX2 (ab76541, abcam), CK20 (ab854, abcam), Ki-67 (ab16667, abcam), LGR5 (ab273092, abcam) and REG4 (ab204171, abcam). For IF the following antibody was used: PD-L1 (GE006, DAKO omnis). Images were acquired by EVOS FLAUTO2 (Thermo Fisher Scientific).

### Isolation and culture of immune cells

T-cells and Myeloid Derived Suppressor Cells (MDSCs) were isolated from patient peripheral blood mononuclear cells (PBMCs) by CD3^+^ immunomagnetic negative selection (#17951, StemCell Technologies) and CD33^+^ immunomagnetic positive selection (#17876, StemCell Technologies) following positive selection with CD3^+^ immunomagnetic positive selection (#17851, StemCell Technologies), respectively.

T-cells were cultured in RPMI medium supplemented with 10% FBS (10270106, Gibco), 1% L-Glutamine (25030-081, Gibco), 1% Hepes, 10 ng/ml IL-2 (202-IL, R&D Systems) and 10 ng/ml IL-7 (207-IL, R&D Systems); MDSCs were cultured in RPMI medium supplemented with 10% FBS, 1% L-Glutamine, 1% Hepes and 10 ng/ml GM-CSF (GFH8AF, Cell guidance systems) for 7 days, then medium was replaced with organoid culture medium for 72 h. The MDSC^CM^ was harvested and stored frozen until use in the PDO culture or in the T-cell culture.

### RT-PCR

Total RNA was isolated following manufacturer instructions (AM1561, Invitrogen, Thermo Fisher Scientific) and complementary DNA (cDNA) was obtained using High Capacity cDNA Reverse Transcription Kit (4368813, Thermo Fisher Scientific). For RT-PCR, expression of MDSCs markers (ARG1, TGFb, IL10, CCL4, IDO1, NOS2), tolerogenic genes (LGALS1. LGALS3, LGALS9, MUC1 and MUC2) and REG4 were assessed using 10 ng of cDNA per sample following Power Up SYBR Green Master Mix instructions (A25742, Thermo Fisher Scientific).

### Priming of T-cells

Patient T-cells were educated to recognize tumor antigens by culturing them in the presence of 500 U/ml IFNγ (GFH77AF, Cell guidance system) pre-treated tumor organoids for 48 h. Then, cells were harvested and gently layered on top of the Ficoll-Paque™ PLUS (17-1440-02, GE Healtcare) and centrifuge at 650rcf for 30 min. T-cells appeared as a ring on Ficoll layer, they were collected with a disposable Pasteur, washed with PBS and finally labeled with 500 nM CMPTX dye (C34552, Invitrogen) according to manufacturer’s instruction, prior to being used in the co-culture.

### Immunosuppression assay

Immunosuppressive potential of the conditioned media from MDSCs was evaluated by CFSE assay. Briefly, T-cells were isolated from healthy donors’ PBMCs, marked with CFSE dye (C34554, Invitrogen) and plated 50000 cells/well in 96-well plate, in the presence of T-cell culture medium or MDSC conditioned media and stimulated with CD3/CD28 beads (11161D, Gibco). CFSE concentration was evaluated by FACS analysis.

### Ex vivo patient-derived immunity-organoid interaction platform establishment

PDOs were plated in the xeno-free matrix Vitrogel (VHM03, The WellBioScience) according to manufacturer’s instructions at the concentration of 500 organoids/plate in 48-well plate. PDOs were successively treated with 500 nM CMPTX-marked T-cells (Effector:target ratio 200:1), MDSCs conditioned medium and/or 20 µg/ml pembrolizumab (anti-PD-1) for 48 h. Apoptosis induction was evaluated by acquiring CellEvent™ Caspase-3/7 Green ReadyProbes™ Reagent (R37111, Invitrogen) fluorescence with EVOS FLAUTO2 (Thermo Fisher Scientific) and measured with ImageJ-Fiji software (National Institutes of Health, Bethesda).

### ELISA assay on conditioned medium

The conditioned medium was analyzed for Granzyme B (GZMB) using Luminex XMAP technology according to manufacturer’s instructions (Bioplex 200, Bio-Rad). Compound concentration in samples was determined from the standard curve using a five-point regression.

### Multiplex Immunofluorescence Analysis

Whole 5 µm tissue sections were dewaxed and rehydrated. Hematoxylin and Eosin staining was performed according to standard protocols. We performed multiplex IF analysis by the Opal 6-Plex Detection Kit (Akoya Biosciences) following standard protocol.

The following antibodies were used for IF analysis: PANCK (M3515, DAKO omnis), REG4 (40321, Signalway), MUC1 (ab109185, Abcam), MUC5AC (ab3649, Abcam), CD4 (ab288724, Abcam), FOXP3 (ab20034, Abcam) CD8 (ab251597, Abcam), GZMB (46890, CST), CD68 (MA5-12407, Invitrogen). Before proceeding, optimal staining conditions for each marker were determined using monoplex stained slides from positive control for each antibody. Multiplex slides images were acquired by Phenoimager Workstation (Akoya Biosciences) and processed with QuPath for cell segmentation and positive cell count.

### Statistical analysis

Each experiment was performed in at least three biological replicates. The F-test was used to estimate the variances between groups. Non-parametric tests, such as the Wilcoxon or Kruskal-Wallis test, were used to estimate differences between paired and multiple groups, respectively. All *p*-values were corrected for multiple testing using the False Discovery Rate (FDR).

## Results

### The MSS CRC group exhibits heterogeneous immunological features

**Fig. 1:**
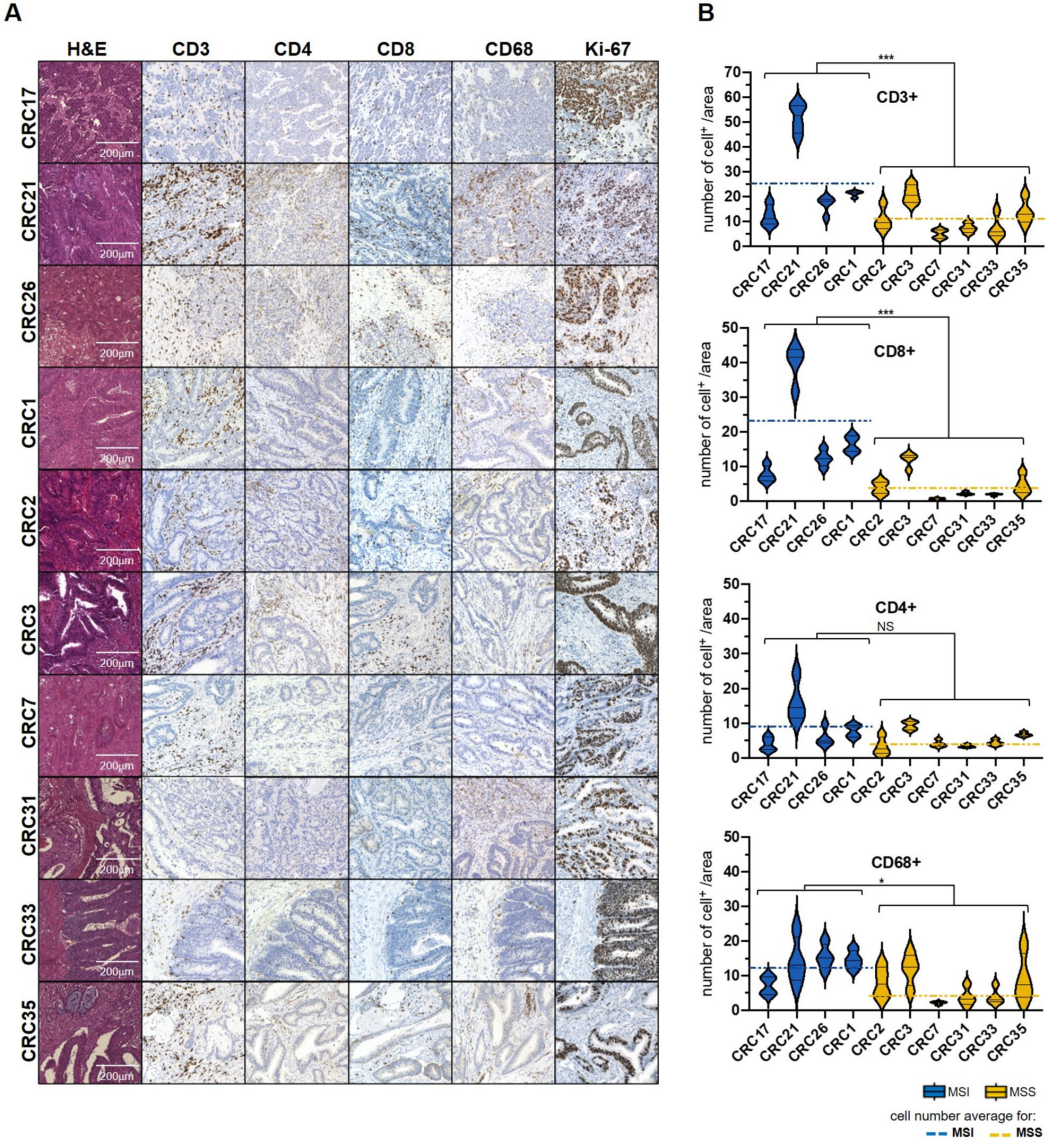
Assessment of immune infiltration in tissue samples from MSI and MSS CRC patients. **A** Immunohistochemical staining of CD3, CD4, CD8, CD68 and Ki-67 in CRC tissues showed the presence of T-cells populations (CD4^+^, CD8^+^) and macrophages (CD68^+^) in MSI and MSS patients. Ki-67 was used as a control proliferation marker of tumor cells; (**B**) Quantification of positive cells/area was performed with QuPath Software. Significance shown refers to Wilcoxon test *p*-values *< 0.05, **< 0.01, ***< 0.001.

To investigate the mechanisms underlying resistance to immunotherapy, we developed organoids cultures from fresh tissue specimens of CRC patients. We performed oncological genomic profiling of tissue samples from 129 patients undergoing surgery at our Institution in 2021-2022 and assessed mutational profiles, microsatellite stability status and tumor mutational burden (TMB) (TruSight Oncology 500, Illumina) (Supplementary Table 1). Consistent with current literature [30], the majority of samples were MSS, while 11 patients exhibited a MSI molecular profile. We established patient-derived organoids (PDOs) from 6 MSS and 4 MSI patients with available fresh tissues and collected matched blood samples. PDOs were maintained in culture for at least one month under selective condition to prevent normal cell contamination. IHC analysis of surgically resected primary tumors revealed that MSI tumors had a higher overall number of infiltrating CD3^+^ T-cells, CD8^+^ T-cells and CD68^+^ cells compared to MSS tumors (Fig. 1A, B). However, we observed diversity in the extent of immune infiltration within MSS tumors, with some of them exhibiting classic MSS features, from here on named MSS type I tumors, whereas others displaying immune infiltration patterns resembling those of MSI tumors, from here on named MSS type II tumors.

**Fig. 2:**
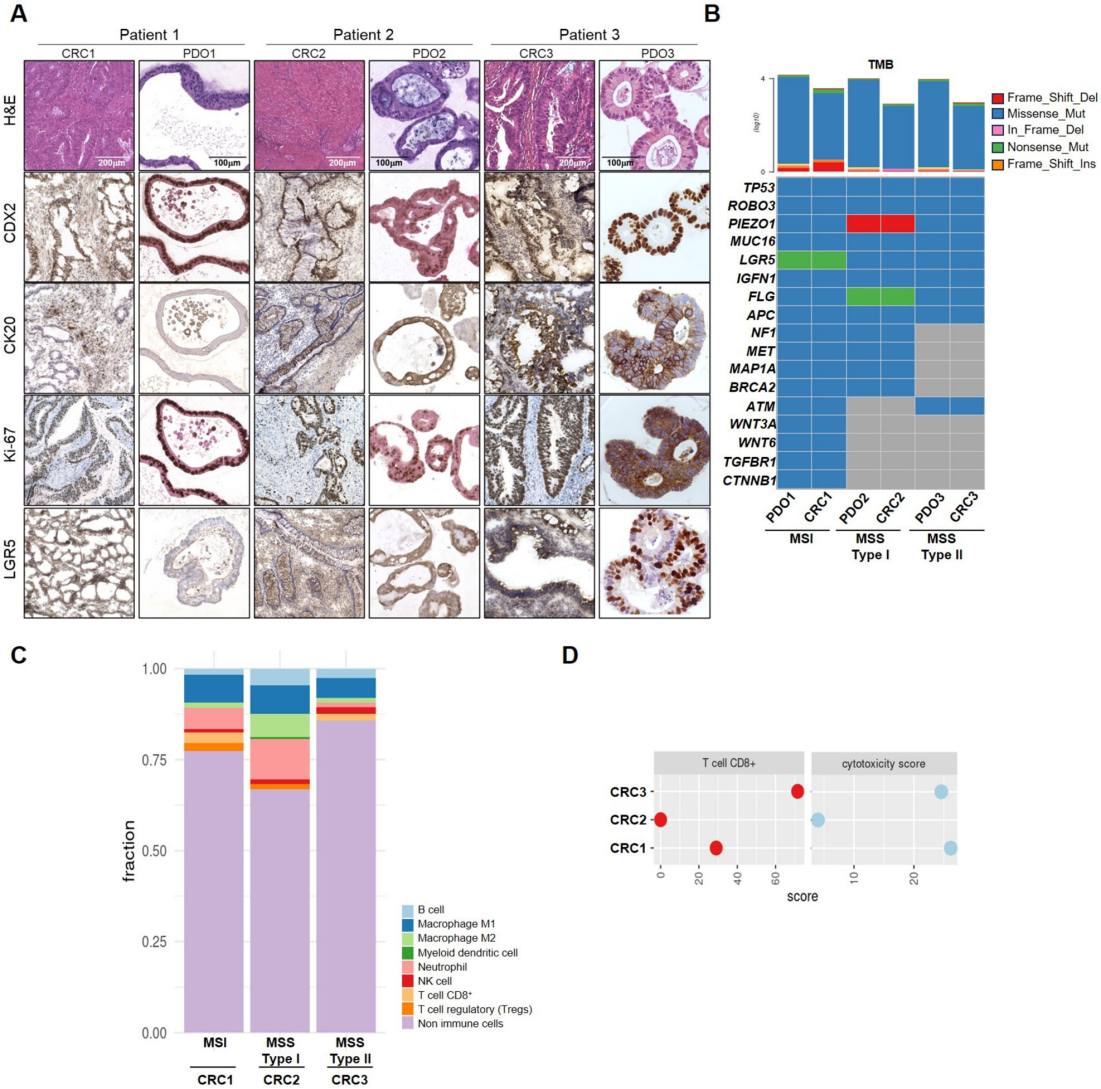
PDOs recapitulate the histopathological and genomics characteristics of their primary tumors. **A** Immunohistochemical staining of CRC specific markers (CDX2, CK20, Ki-67, LGR5) confirmed matching between organoids (PDO) and tissue samples (CRC). **B** Lower panel, Oncoplot showing the top 25 mutated genes in PDOs and matched samples identified by WES. Upper panel, bar plot shows the tumor mutational burden (TMB). **C** The Bar plot displays the ratios of immune cell populations, determined using the immunedeconv package with the quantiseq algorithm. **D** Dotplots showing the immunodeconvolution scores for CD8^+^ T-cells and Cytotoxicity estimated with the Microenvironment Cell Populations (MCP) counter algorithm.

Next, we characterized three PDO models representative of each identified group: PDO1 (MSI model), PDO2 (MSS type I model), and PDO3 (MSS type II model) along with the matched primary tumors. PDOs closely recapitulated the histopathological and genomics characteristics of their corresponding primary tumors (Fig. 2). The tumor origin of PDO cultures and the absence of healthy organoid overgrowth were confirmed by CRC markers staining (Fig. 2A). Whole-Exome Sequencing (WES) analysis confirmed that organoids retained the mutational profile of the original tumors (Fig. 2B). As expected, MSI CRC exhibited a higher TMB compared to the other models in both organoid and tumor tissue specimens (Fig. 2B). Consistent with the previously described IHC analysis, transcriptomic and immunodeconvolution analysis demonstrated that MSS type II CRC tissue has a tumor infiltrating immune profile akin to that of the MSI tumor (Fig. 2C).

To further assess the immune features of these tumors, we applied the Microenvironment Cell Populations (MCP) counter algorithm on transcriptomic data to calculate the cytotoxicity score, reflecting the immune system’s capacity to recognize and eliminate tumor cells. Notably, MSS Type II tumor closely resembles the MSI tumor, exhibiting higher cytotoxicity score and CD8^+^ T-cells infiltration compared to the MSS Type I tumor (Fig. 2D).

Overall, these data demonstrate that the MSS tumors may exhibit intragroup heterogeneity, and some of them may share phenotypic characteristics with MSI tumors, suggesting potential sensitivity to Immune Checkpoint Inhibitors (ICIs).

### Ex vivo CRC patient-derived immuno-organoids (PD-IOs) interaction platform unveils that MSS type II CRC may respond to immunotherapy

To study immunotherapy response in a complex system that recapitulates human pathology, we developed a patient derived immuno-organoids (PD-IOs) interaction platform with patient-matched T-cells, MDSCs conditional medium (MDSC^CM^) and tumoral PDOs in absence or presence of anti-PD-1 (pembrolizumab). This platform not only provides advanced and flexible ex vivo study model but also addresses the scarcity of MSS CRC patient samples treated with immunotherapy, which is not currently an approved therapy for this group.

**Fig. 3:**
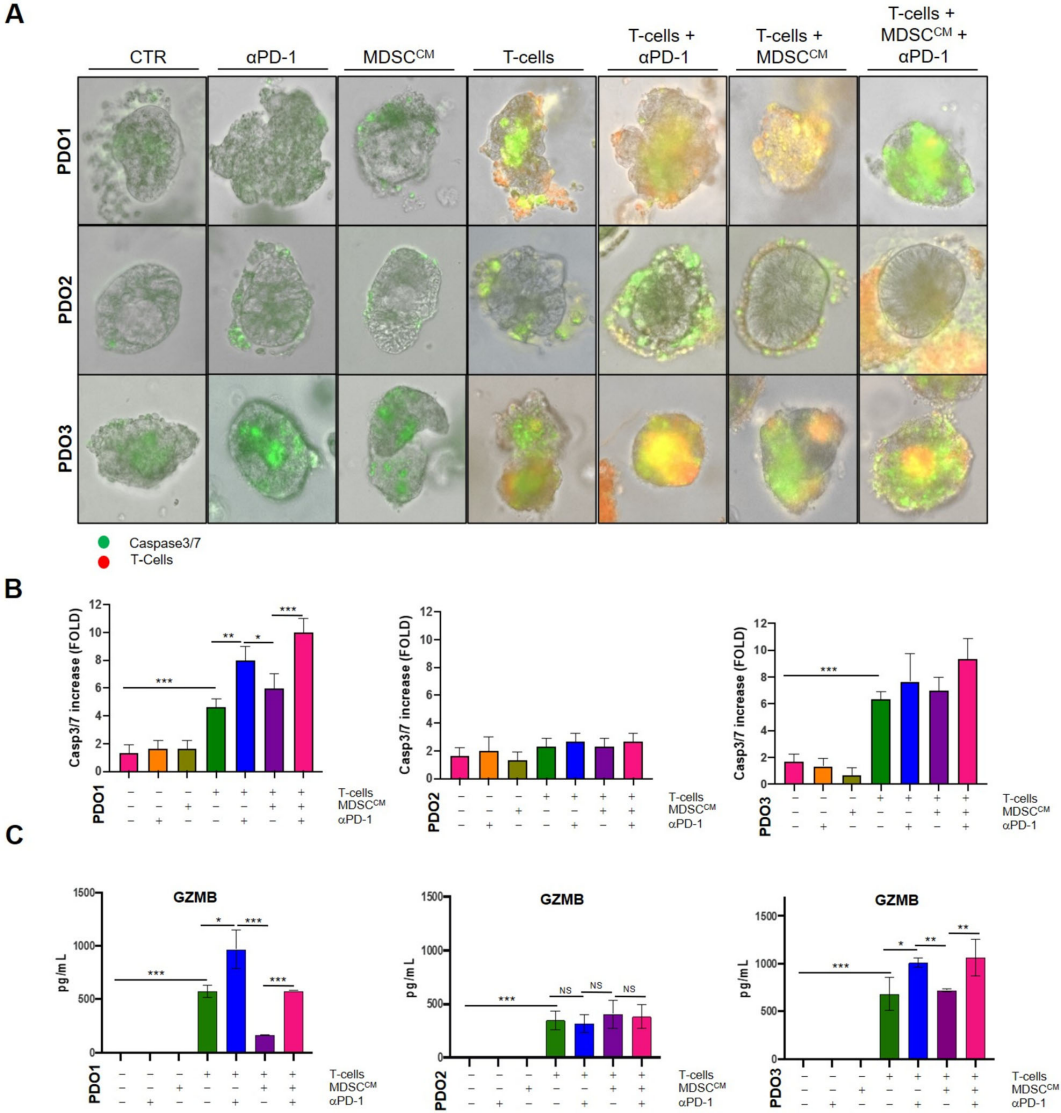
PDOs showed different sensitivity to T-cells in ex vivo interaction platform according to cytotoxicity score. **A** Immunity-organoid interaction platforms with patient-matched T-cells, MDSCs and PDOs in absence or in presence of pembrolizumab (anti-PD-1). Caspase 3/7 activation was measured by Cell Event Caspase 3/7 Green ReadyProbes Reagent (Invitrogen) fluorescence to evaluate apoptosis induction with Evos FL Auto 2 (Thermo Fisher Scientific) and (**B**) measured with ImageJ-Fiji Software. **C** T-cells’ GZMB release was evaluated by Luminex XMAP technology and correlates with the measured apoptosis induction. Significance shown refers to Kruskal-Wallis test *p*-values *< 0.05, **< 0.01, ***< 0.001.

Initially, we verified that PDOs maintained their original tissue characteristics through immunostaining and WES (as previously described in Fig. 2A, B), including PD-L1 expression (Supplementary Fig. S1). T-cells (CD8^+^ and CD4^+^) isolated from peripheral blood mononuclear cells (PBMCs) were expanded and characterized for activation markers (CD25, HLA-DR), PD-1 expression (CD279) and proliferative capacity (Supplementary Fig. S2A, B). Freshly isolated MDSCs were cultured in vitro and conditioned media (MDSC^CM^) were collected for the subsequent steps. MDSCs were characterized by RT-PCR for TGF-β and IL-10 expression (Supplementary Fig. S2C), as well as for myeloid markers ARG1, NOS2, IDO1 and CCL4 (Supplementary Fig. S2D) and their capacity to suppress the activation of T-cells isolated from a healthy donor (Supplementary Fig. S2E). Consistent with the previous discussed results, MDSC^CM^ from MSS CRC patients showed different immunosuppressive features. Notably, MDSCs isolated from the CRC3 patient displayed an immunosuppressive ability more similar to that of the MSI CRC patient. Finally, the organoids were engaged with T-cells, previously activated and stained, in the presence or absence of the immunosuppressive stimuli secreted by MDSCs (MDSC^CM^) and the ICI pembrolizumab (Fig. 3). We assessed apoptosis induction of organoids using CellEvent™ Caspase-3/7 Green ReadyProbes™ and time-lapse live microscopy.

As expected, T-cells were unable to recognize tumor cells in the MSS type I PDO2 platform, reflecting the immune desert landscape characteristic of classical MSS CRC. On the contrary, autologous T-cells induced apoptosis of the MSI PDO1 model with a further increase after 48 h of pembrolizumab treatment. Interestingly, MSS type II PDO3 platform exhibited features close to those observed in the MSI with a baseline increase in T-cell recognition and cytotoxic activity, as well as an enhanced release of pembrolizumab-induced Granzyme B (GZMB) in the co-culture conditioned media, corroborating previous cytotoxicity score data (Fig. 3A–C). Despite MDSCs exhibit an immunosuppressive ability on T-cells from healthy donors, the addition of MDSC^CM^ has less pronounced effect in this type of platform.

By utilizing a cutting-edge ex vivo interaction tool, these findings confirm the existence of a subgroup of MSS CRC tumors that are more prone to be affected by immune activation strategies.

### Transcriptome analysis reveals cancer-specific markers (CST) of resistance to immunotherapy

**Fig. 4:**
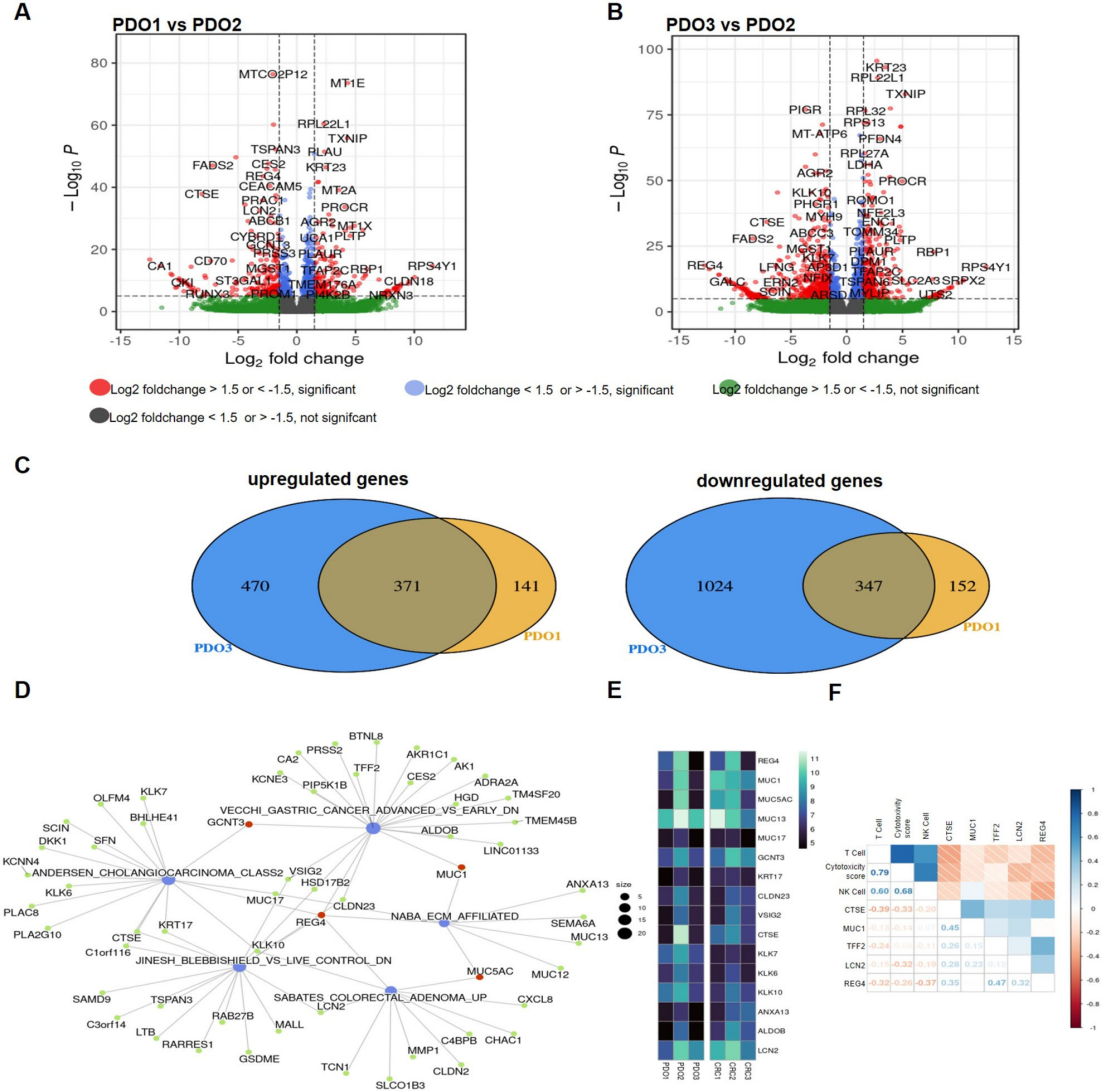
Transcriptomics analysis. Volcano plot showing differentially expressed genes between (**A**) PDO1 and PDO2 and (**B**) PDO3 and PDO2. **C** Venn Diagram showing common differentially expressed genes in PDO1 (MSI) and PDO3 (MSS Responder) compared to PDO2 (MSS Non-Responder). **D** Network Plot showing the role of REG4 as hub gene in the common downregulated genes. **E** Heatmap showing expression of REG4 oncogenic signature in PDOs and parental tissues. **F** Plot showing the correlation between REG4 signature genes and T- and NK- cells infiltration and cytotoxicity score estimated by immunodeconvolution in a TCGA CRC patient cohort.

We performed RNAseq analysis to identify the cancer-specific markers (CST) markers associated with CRC resistance to ICI treatment (pembrolizumab). Differentially expressed genes analysis between responder and non-responder PDOs (Fig. 4A, B) highlighted 371 up-regulated and 347 down-regulated common genes in responder PDOs (regardless of microsatellite stability status) compared to the non-responder model (Fig. 4C). Gene Set Enrichment Analysis (GSEA) conducted on the commonly downregulated genes in responder models revealed a specific signature shared with other gastrointestinal tumors, such as cholangiocarcinoma, gastric cancer and colorectal adenoma, with REG4 identified as a hub gene (Fig. 4D, E).

Focusing on CRC patients with high tumor purity from the TCGA database, we found a resistant oncogenic signature of immune evasion (including REG4, CTSE, MUC1, TFF2, LCN2) that inversely correlated with cytotoxicity and immune infiltration deconvolution scores (T- and NK-cells) (Fig. 4F). Accordingly, multiplex immunofluorescence (IF) analysis of our CRC tissues confirmed low to absent expression of REG4 and mucins (MUC1 and MUC5AC) genes in the MSI CRC1 and MSS type II CRC3, while MSS type I CRC2 exhibited positive staining consistent with transcriptomic analysis on PDO models (Supplementary Fig. S3A). Additionally, CRC1 and CRC3 showed a higher proportion of cytotoxic CD8^+^ T-cells (CD8^+^/GZMB^+^) and lower immune-suppressive T-regs (CD4^+^/FOXP3^+^) compared to CRC2 (Supplementary Fig. S3B).

These results suggest the role of the identified hub genes as CST markers associated with impaired infiltration of cytotoxic T-cells, highlighting REG4 and mucin pathways as potential targets to enhance immunotherapy sensitivity.

### Validation of the identified CST markers in a cohort of MSI CRC patients treated with immunotherapy

**Fig. 5:**
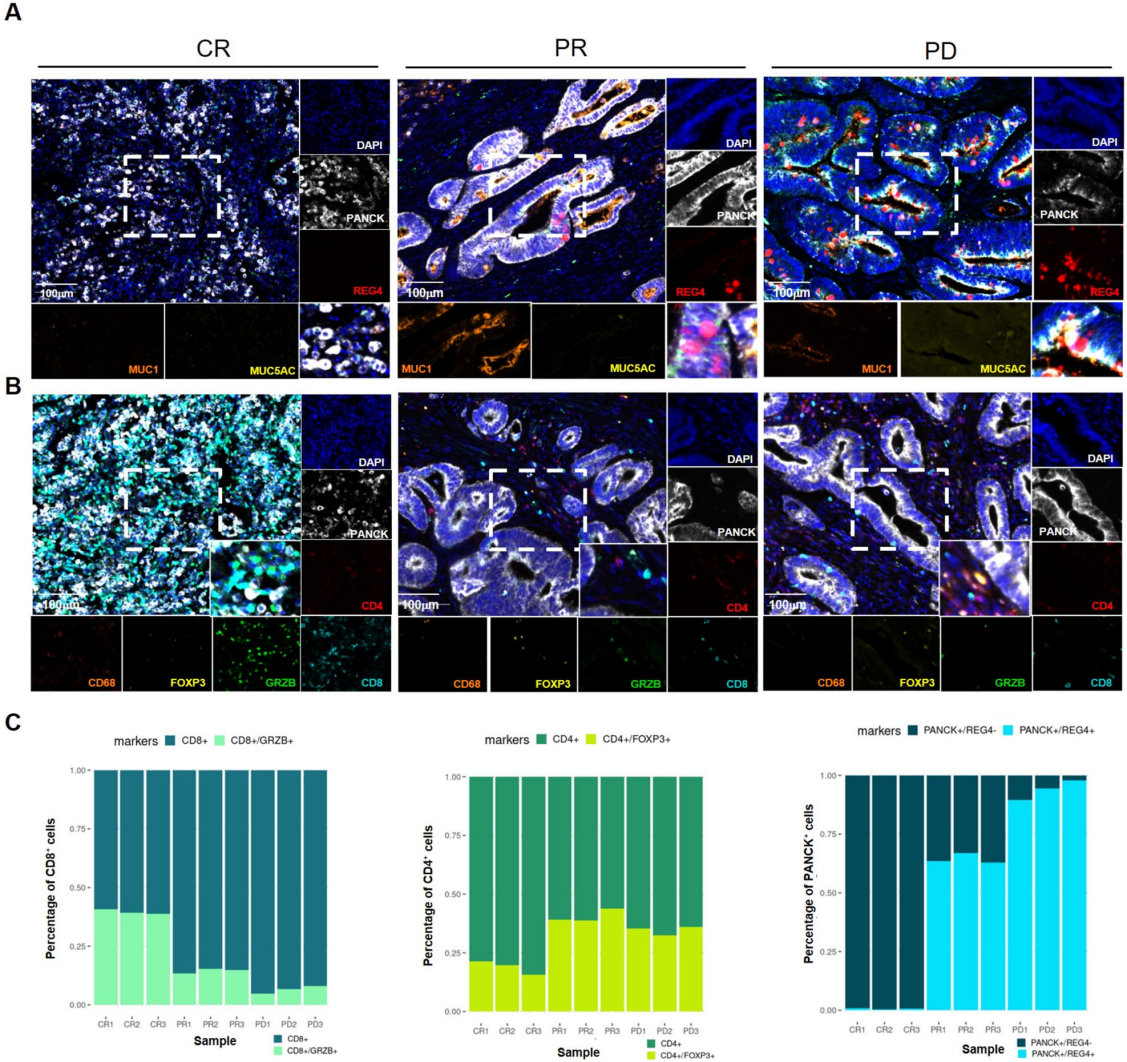
Multiplex immunofluorescence analysis validated REG4 as immunotherapy resistance marker in MSI CRC patient cohort. Adenocarcinoma (NOS) tissues from MSI CRC patients who achieved a complete response (CR), a partial response (PR) or experienced disease progression (PD) after immunotherapy were analyzed by multiplex immunofluorescence to investigate (**A**) PANCK (white), REG4 (red), MUC1 (orange) and MUC5AC (yellow) expression or (**B**) PANCK (white), CD4 (red), CD8 (cyan), GZMB (green), FOXP3 (yellow), CD68 (orange). **C** Graphs showed the percentage of CD8^+^ GZMB^+^/CD8^+^ T-cells (dark turquoise), CD4^+^ FOXP3^+^/CD4^+^ T-cells (green) and PANCK^+^ REG4^+^ /PANCK^+^ cells (blue) in both Mucinous and NOS adenocarcinomas.

To validate our findings, we employed multiplex IF to asses the expression of REG4, MUC1 and MUC5AC in a cohort of MSI CRC patients (n = 9) who underwent immunotherapy-based treatment and achieved either a complete response (CR; *n* = 3), a partial response (PR; n = 3) or experienced disease progression (PD; n = 3) (Supplementary Table S2). The identified CST markers correlated with RECIST response rates in adenocarcinoma CRC patients (Fig. 5A) and in mucinous adenocarcinoma CRC (Supplementary Fig. S4A, C). Tumor tissues analysis for immune infiltration components revealed that PD and PR patients had higher levels of regulatory immunosuppressive T-cells (CD4^+^, FOXP3^+^) compared to CR patients. Conversely, activated T-cells (CD8^+^, GZMB^+^) were more prevalent in tumors from CR patients than in those from PR and PD patients (Fig. 5B and Supplementary Fig. S4B, C).

Notably, MSI CRC tumors of patients who achieved a complete pathological response exhibited low to absent expression of CST markers suggesting that these markers could serve as indicators for immunotherapy response, even in the clinical setting of MSI CRCs, allowing for refined patient selection.

### REG4 mediates immunotherapy resistance

**Fig. 6:**
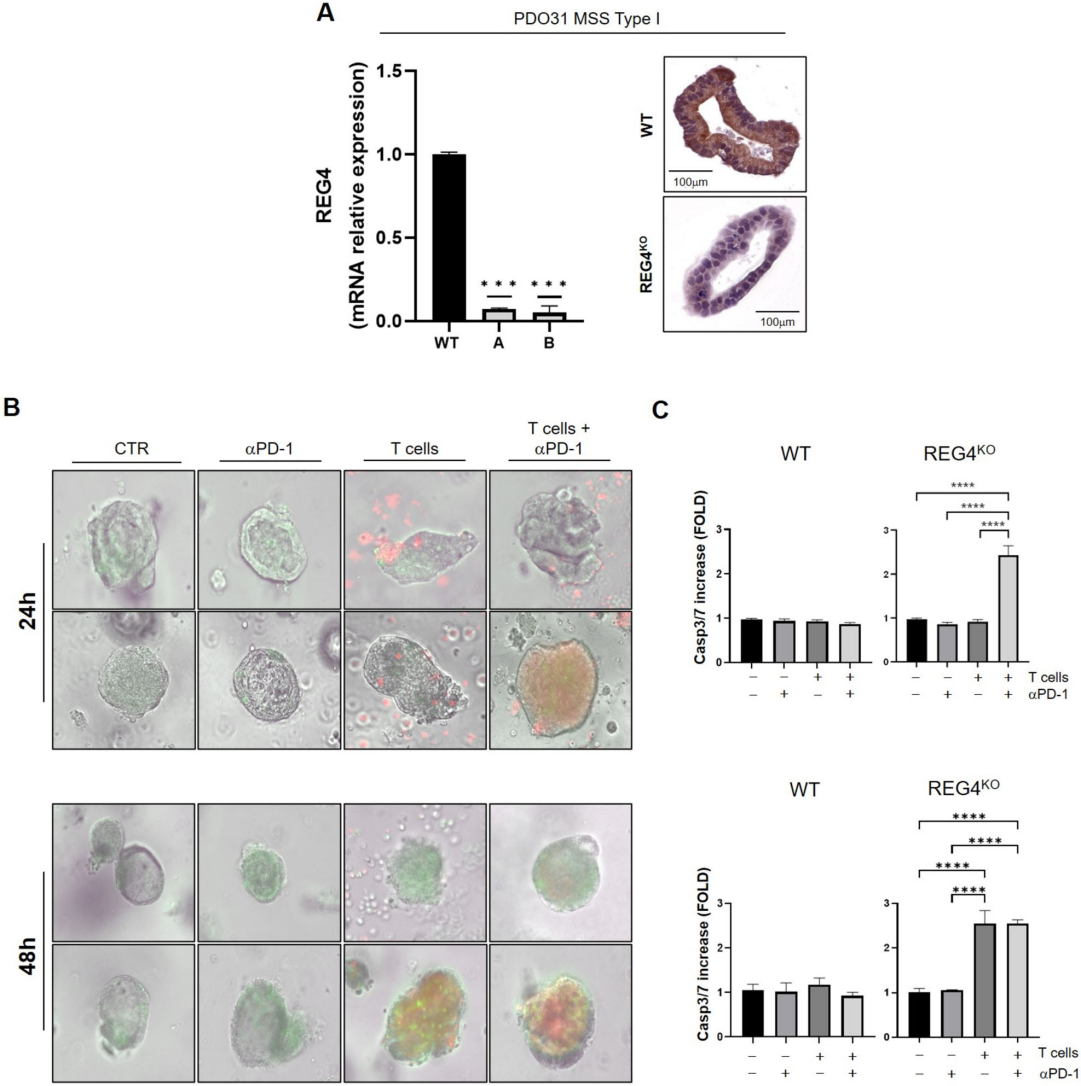
REG4 knock-out restored immune sensitivity. **A** REG4^KO^ in PDO31 was assessed through both RT-PCR and IHC analysis. Significance shown refers to One-way ANOVA test *p*-values ***< 0.001. **B** PD-IOs interaction platforms with patient-matched T-cells and wild-type (WT) or REG4^KO^ PDOs in absence or in presence of pembrolizumab (antiPD-1). Caspase 3/7 activation was measured by Cell Event Caspase 3/7 Green ReadyProbes Reagent fluorescence to evaluate apoptosis induction with Evos FL Auto 2 and (**C**) measured with ImageJ-Fiji Software. Significance shown refers to One-way ANOVA test *p*-values ****< 0.0001.

To investigate whether interfering with REG4 expression would affect tumor ability to evade immune system, we inactivated REG4 gene in type I MSS CRC immunotherapy-resistant PDO model with high REG4 expression (Supplementary Fig. S5A) using small guide RNA (sgRNA) pairs designed to delete the transcription start site. Tumor origin of PDO cultures and the absence of healthy organoids overgrowth were confirmed by CRC tissue markers staining (Supplementary Fig. S5B). Successful gene disruption was confirmed at mRNA and protein levels (Fig. 6A). We subsequently assessed the effect of REG4 knock-out (KO) in the context of our PD-IOs interaction platform using autologous T-cells and PDOs with or without pembrolizumab. Notably, REG4 loss restored T-cells recognition and sensitivity to anti-PD-1 treatment, resulting in a statistically significant increase in apoptosis in treated organoids after 24 h of co-culture and enhanced basal T-cells cytotoxicity by 48 h (Fig. 6B, C). Moreover, REG4^KO^ organoids showed reduced expression of tolerogenic molecules, such as galectins (LGALS1, LGALS3, LGALS9) and mucin (MUC1, MUC2) compared to the wild-type counterpart highlighting the potential mechanism for REG4-dependent immune evasion (Supplementary Fig. S5C).

Collectively, these results suggest that REG4 promotes the expression of ligands that impair T-cell cytotoxic activity, and its inhibition enhances antitumor immune reactivation and anti-PD-1 treatment efficacy.

## Discussion

In this study, we have unveiled novel cancer specific tissue (CST) markers of resistance to immunotherapy in CRC patients. Using a clinically relevant ex vivo CRC Patient Derived Immuno Organoids (PD-IOs) interaction platform, we were able to recapitulate the complex interaction between tumor and the most relevant immune cell components of tumor microenvironment (TME). This advanced system enables ex vivo study of the pharmacological perturbation of the interaction between tumor and immune system, as well as mechanisms of immunotherapy resistance, approaches that would otherwise be only hypothetically conceivable in patients not eligible for immunotherapy, such as the MSS subgroup.

Immunotherapy has been demonstrated as an effective weapon for treating various types of tumors [9, 10]. In the context of CRC, this approach is particularly effective in treating MSI tumors, which constitute only a fraction (15%) of all CRC cases [31, 32]. In 2020, the FDA approved pembrolizumab as a first-line treatment option for metastatic colon tumors with MSI [https://www.fda.gov/drugs/drug-approvals-and-databases/fda-approves-pembrolizumab-first-line-treatment-msi-hdmmr-colorectal-cancer]; despite this, about 50% of MSI CRC patients still exhibit resistance to the drug [15, 33]. Compared to patients with MSI CRC, those with MSS disease generally show weaker responses to ICIs. However, remarkable responses have been noted even in a subset of MSS patients [16, 30]. Therefore, it remains of primary importance to identify the resistance markers associated with ICI treatment to improve patient selection for MSI subgroup and to enlarge the cohort of patients who could benefit from immunotherapy by better selecting the potential MSS responder patients and, finally, to discover new therapeutic targets.

Our study stems from a genetic screening of a large cohort of CRC patients followed at our institution. The tumors from these patients were categorized based on microsatellite stability status rather than utilizing the more recent Consensus Molecular Subtypes (CMS) classification [34]. We reasoned that while the CMS classification proved effective for prognostic purposes, it did not provide practical guidance for selecting therapeutic approaches.

To overcome the impossibility of studying the effects of ICIs resistance on patients who currently cannot benefit from immunotherapy, we established a platform between PDOs and autologous components of the immune system to recreate simplified ex vivo interactions. Since secreted factors from MDSCs can mediate inhibitory signal on T-cells [8, 35] we included conditioned media from freshly isolated MDSCs in our platform [34]. Therefore, we recreated the interaction between the tumor (in the form of organoids) and two main immune components, T-cells and MDSCs. We recognize that this system is certainly a simplification of the complex interplay between the tumor and the TME [25]. However, to the best of our knowledge, it is one of the most accurate integrated models for studying these interactions. Our team has previously utilized other preclinical models, such as NSG-immune-humanized mice [36] and PDX models [37, 38], but this is undoubtedly the model with fewer issues and higher reproducibility.

Furthermore, we acknowledge that the MDSCs used in this study represent an enrichment of CD33^+^ myeloid cells (including both the monocytic and polymorphonuclear fractions) and may be a limited and mixed representation of immunosuppressive cell populations. To better define these cellular subtypes, we referred to the most comprehensive characterizations available in the literature [39] and their immunosuppressive ability. However, this immunosuppression appears significantly different between T-cells from healthy donors and T-cells from the same patient. This diversity might stem from defects in T-cells after prolonged exposure to the tumor or from the generally suppressive systemic environment of the tumor. Nevertheless, this study does not aim to focus on the immunosuppressive effects of MDSCs on T-cells, but rather intends to recreate an in vitro environment that closely resembles that of the patient, while acknowledging the limitations and simplifications of this model.

Another crucial aspect of our platform is the use of immune system components derived from patient’s peripheral blood. While this might seem like a limitation at first glance, it is actually a significant strength. Our system is focused on the interaction of the tumor with an immune system that is still active and not compromised by the proximity of the tumor and thus by the long exposure to its suppressor influence. Drawing from peripheral blood rather than the TME allowed us to circumvent issues related to the low presence of T-cells in immune desert tumors or compromised immune activation due to exhaustion [40, 41].

From the study of the platform and the perturbation of the interaction through anti-PD-1, we demonstrated that even some MSS tumors, fitting with specific characteristics, might be sensitive to immunotherapy, providing the opportunity to delve into the mechanisms behind anti-PD-1 resistance. Differential expression analysis (DEA) between responder and non-responder organoids uncovered that the gastrointestinal oncogenic REG4 signature is inversely correlated with CD8^+^ T-cells infiltration and activation.

REG4 is widely expressed in gastrointestinal tumors and usually defines cancer subtypes with intestinal and goblet-like differentiation and with poor prognosis [42]. REG4 operates downstream the transcription factor GATA6 [43] and has shown potent mitogenic and pro-metastatic effects in gastric and colon cancers [44, 45]. Here for the first time, we showed the association of REG4 positivity with impaired immune activity and immunotherapy resistance in CRC.

Moreover, we found that the REG4 positive CRC tumors also expressed high levels of MUC1 and MUC5AC, other two minor hub genes of the gastrointestinal oncogenic REG4 signature. These glycoproteins play an important role in protecting the epithelia of most organs from physical and chemical damage and infection [46]. However, the anomalous expression of these proteins has been associated with promoting tumor growth, progression and metastasis [47]. Studies have indeed shown that these proteins contribute to the formation of an immunosuppressive TME [48, 49], which prevents immune cells from recognizing and attacking tumor cells. Specifically, aberrant mucin glycosylation on cancer cells leads to the expression of atypical epitopes, resulting in the recognition and binding of cancer cell membrane glycosylation patterns that trigger apoptosis of cancer-specific effector T-cells [47]. Moreover, our results demonstrated that knock out of REG4 reduced the expression of MUC1 and galectins, other molecules with immunosuppressive properties [50–52], pinpointing the activation of a secreted tolerogenic program as potential REG4-mediated immune evasion mechanisms.

The gastrointestinal oncogenic REG4 signature is shared from both NOS (not otherwise specified) and mucinous adenocarcinoma. The latter is a distinct form of colorectal cancer which represents at least 10% of patients with a CRC diagnosis [53, 54] often associated with a poor prognosis in the metastatic setting, with shorter progression-free and overall survival and decreased responsiveness to systemic chemotherapy compared with adenocarcinoma [55, 56]. Moreover, mucinous colorectal cancer is more frequently associated with MSI than NOS adenocarcinoma [57].

Although there are some limitations in this study, the identified markers offer a more dependable means to forecast the responsiveness of CRC patients to anti-PD-1 treatment, going beyond the microsatellite status. Additionally, uncovering markers of immune resistance may pave the way for the development of novel therapies with the potential to facilitate an immunogenic shift in this particular subset of tumors, rendering them responsive.

The results of our study suggest REG4 as a new immunotherapy resistance predictive factor that could be further investigated in future clinical trials with a biomarker-driven selection of patients. These findings will pave the way to enhance the selection of MSI patients who could benefit from immunotherapy. Moreover, the achievements on the resistance marker herein identified may be translated also in MSS CRC patients, accounting for 85% of all CRC cases, exploring the likelihood of a shared marker of treatment resistance.

## Supplementary information

Supplementary Figure Legends

Supplementary Fig. S1

Supplementary Fig. S2

Supplementary Fig. S3

Supplementary Fig. S4

Supplementary Fig. S5

Supplementary Table S1

Supplementary Table S2

## Data Availability

The TSO500 data are available upon request. RNAseq data are uploaded onto Zenodo platform (10.5281/zenodo.14203705).
