# Supplementary material for: Colorectal cancer patients-derived immunity-organoid platform unveils cancer-specific tissue markers associated with immunotherapy resistance
Source: Cell Death Dis. 2024 Dec 4;15(12):878. doi: 10.1038/s41419-024-07266-5 (PMC11618451; doi:10.1038/s41419-024-07266-5)
Supplement: Supplementary file 1 — Supplementary Figure Legends [file 41419_2024_7266_MOESM1_ESM.docx]

**Supplementary Figure Legends**

**Supplementary Fig. S1. PD-L1 was detected in selected patients' tumor tissues and retained in PDO cultures.** Immunofluorescence analysis showed A) the PD-L1 expression in PDOs and matched tumor tissues with B) relative IgG control staining.

**Supplementary Fig. S2. Phenotypical and functional characterization of immune components of the interaction platform.** A) FACS analysis evaluated CD4^+^/CD8^+^ T-cells population and the expression of CD25, HLA-DR (activation markers) and PD-1 (CD279) at the basal status and B) confirmed activated phenotype of T-cells after *ex vivo* expansion and specific tumor priming, whereas T-cells population ratio was retained. C) MDSCs were characterized by RNA expression of cytokines, receptors and D) immune-regulatory molecules measured with RT-PCR. E) CFSE assay showed the immunosuppressive potential of MDSCs conditioned media on healthy donor T-cells in the presence of αCD3-αCD28 beads. Significance shown refers to Kruskal-Wallis test p-values *< 0.05, **< 0.01, ***< 0.001, ****< 0.0001.

**Supplementary Fig. S3. Multiplex immunofluorescence analysis validated gastrointestinal oncogenic REG4 signature in CRC tissues samples.** A) Multiplex IF on CRC primary tissues for PANCK (white), REG4 (red), MUC1 (orange), MUC5AC (yellow). B) Immune profiling by IF on CRC primary tissues. The markers used are PANCK (white), CD4 (red) FOXP3 (yellow), CD8 (cyan), GZMB (green), CD68 (orange). C) Graphs showing the percentage of CD8^+^ GZMB^+^/CD8^+^ T-cells (dark turquoise), CD4^+^ FOXP3^+^/CD4^+^ T-cells (green) and PANCK^+^ REG4^+^ /PANCK^+^ cells (blue).

**Supplementary Fig. S4. MSI CRC patient cohort multiplex IF additional data.** A) Exemplificative images of mucinous tissues from MSI CRC patients who achieved a partial response (PR), or experienced disease progression (PD) after immunotherapy were analyzed by multiplex immunofluorescence to investigate A, PANCK (white), REG4 (red), MUC1 (orange) and MUC5AC (yellow) expression or B) PANCK (white), CD4 (red), CD8 (cyan), GZMB (green), FOXP3 (yellow), CD68 (orange). C) Boxplots showing the positive cells (normalized for area) of each marker in all patients of CRC MSI validation cohort showing complete response (CR), partial response (PR) and progression of disease (PD). Significance shown refers to Wilcoxon test, p-values *< 0.05, **< 0.01, ***< 0.001.

**Supplementary Fig. S5. Validation of REG4^KO^ and its effects on mucins and galactins.** A) REG4 mRNA expression in MSS type I models (PDO2 and PDO31), MSS type II model (PDO3) and MSI model (PDO1). B) Immunohistochemical staining of CRC specific markers (CDX2, CK20, Ki-67, LGR5) confirming matching between PDO31 and tissue sample CRC31. C) Galectin and mucin mRNA expression levels in PDO31 W.T. and REG4^KO^. Significance shown refers to t-test p-values *< 0.05, **< 0.01, ***< 0.001.

**Supplementary Table S1.** **Mutational profiles and Microsatellite stability status of CRC patients.** The table reports the mutation identified by TSO500 and microsatellite stability status.

**Supplementary Table S2. Clinical information of MSI CRC patients retrospective cohort.** The table reports immunotherapy treatment response and histological tumor features in MSI CRC patients of the validation cohort. In particular, histotype (mucinous or NOS - Not Otherwise Specified), tumor grading and staging, the presence of budding and of perineural infiltration (PNI) are described. NV indicates data not valuable.
