# Supplementary figures and images for: Colorectal cancer patients-derived immunity-organoid platform unveils cancer-specific tissue markers associated with immunotherapy resistance
Source: Cell Death Dis. 2024 Dec 4;15(12):878. doi: 10.1038/s41419-024-07266-5 (PMC11618451; doi:10.1038/s41419-024-07266-5)

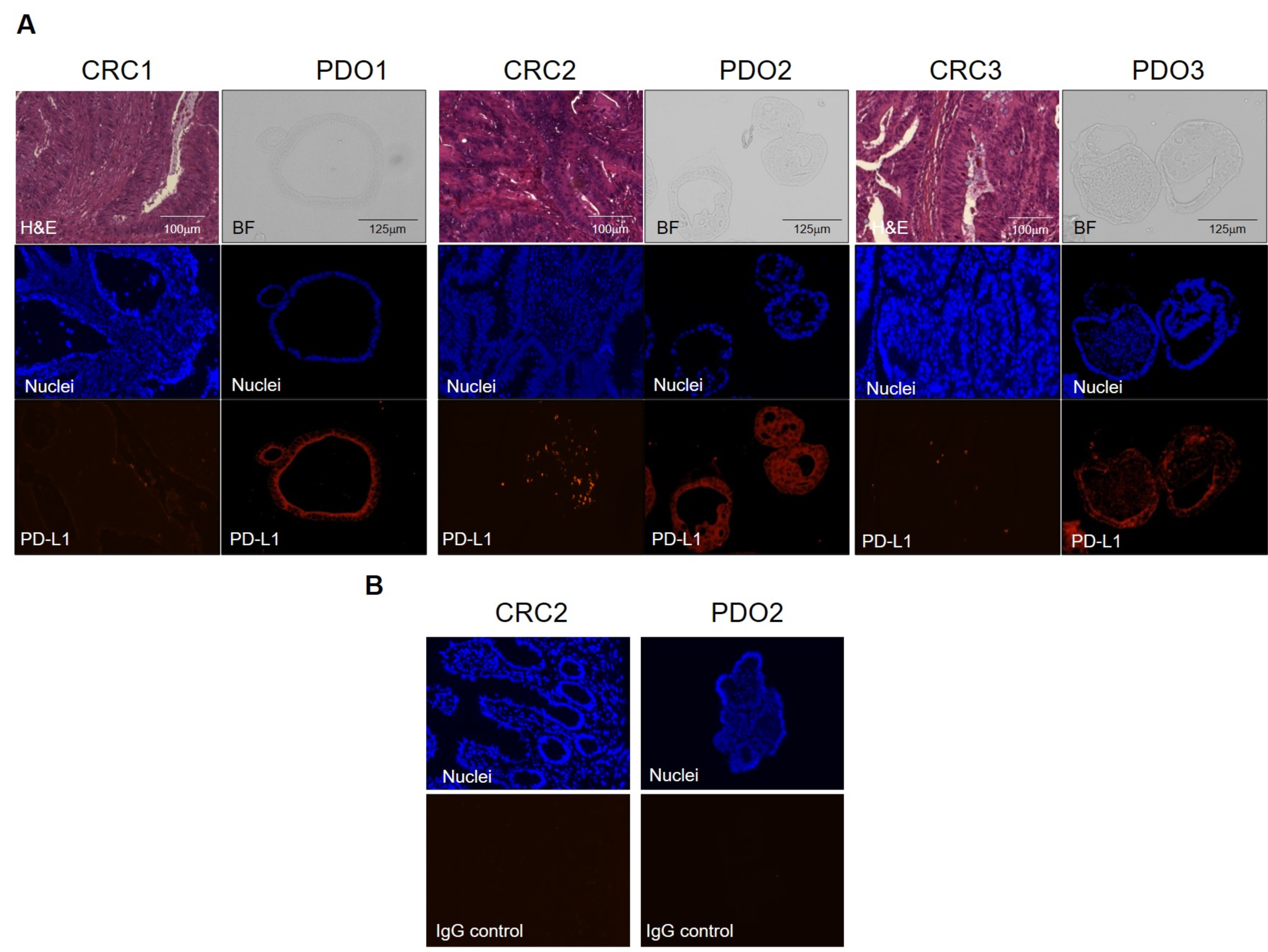

Supplement: Supplementary file 2 — Supplementary Fig. S1 [file 41419_2024_7266_MOESM2_ESM.jpg]

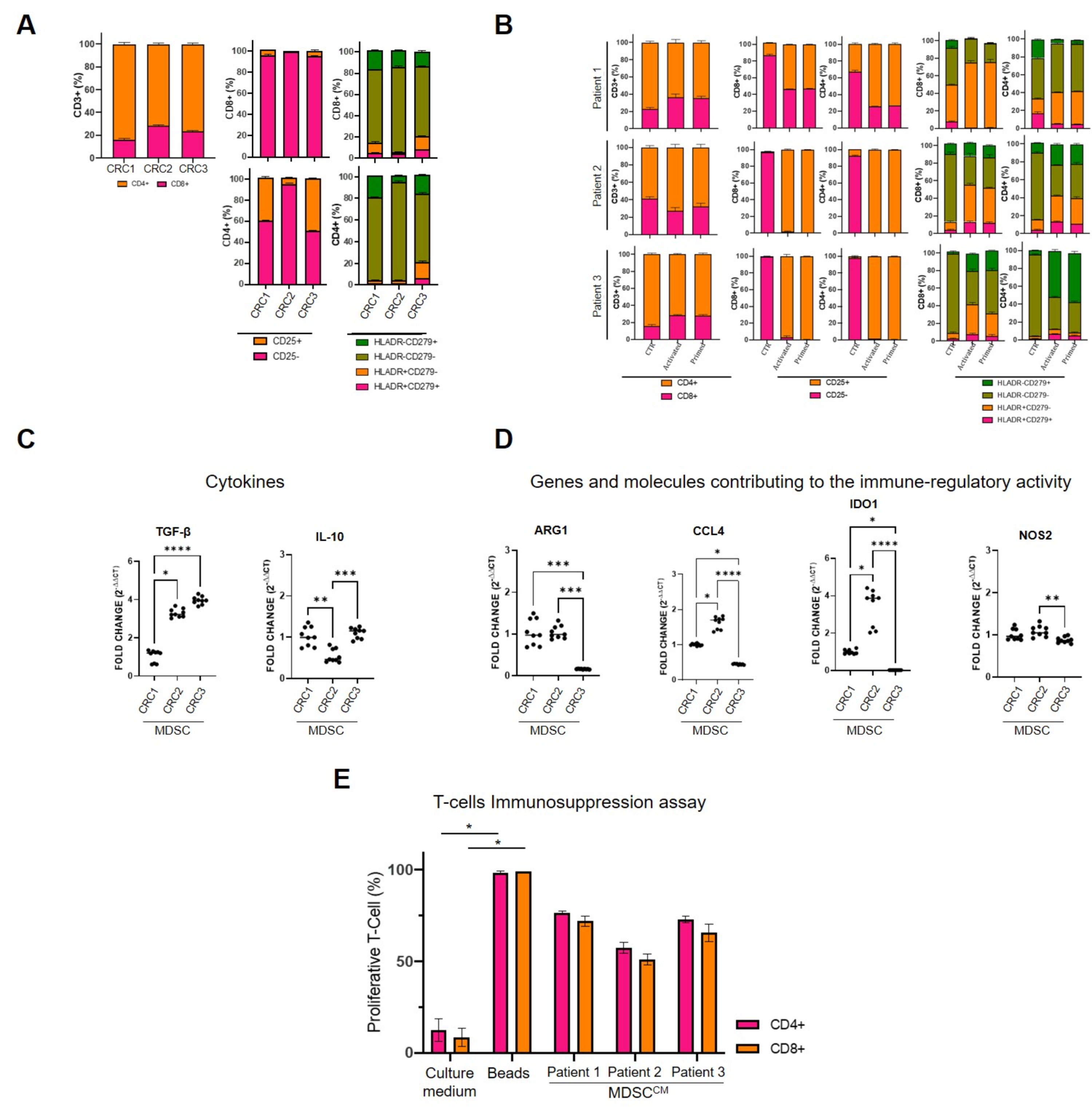

Supplement: Supplementary file 3 — Supplementary Fig. S2 [file 41419_2024_7266_MOESM3_ESM.jpg]

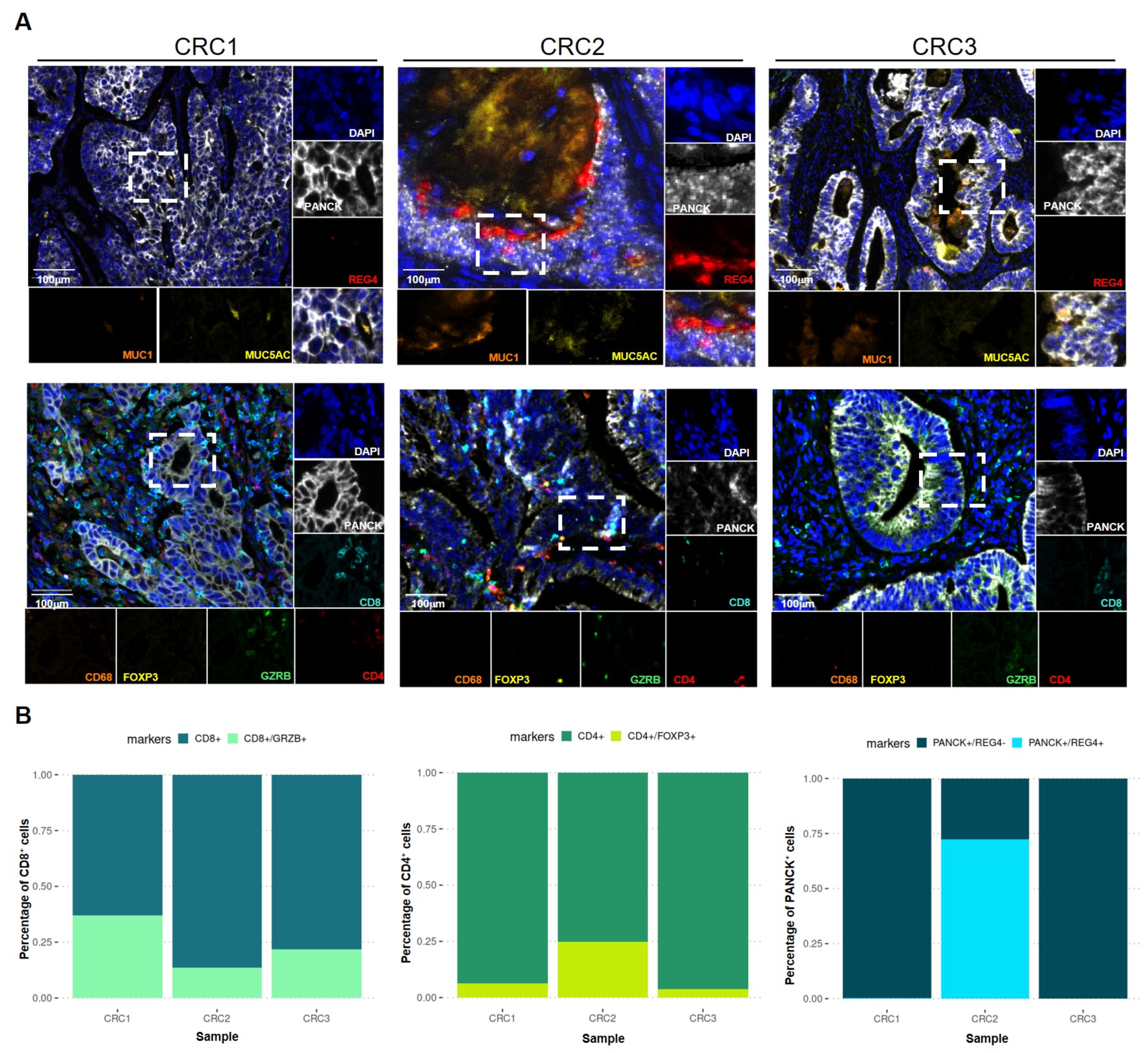

Supplement: Supplementary file 4 — Supplementary Fig. S3 [file 41419_2024_7266_MOESM4_ESM.jpg]

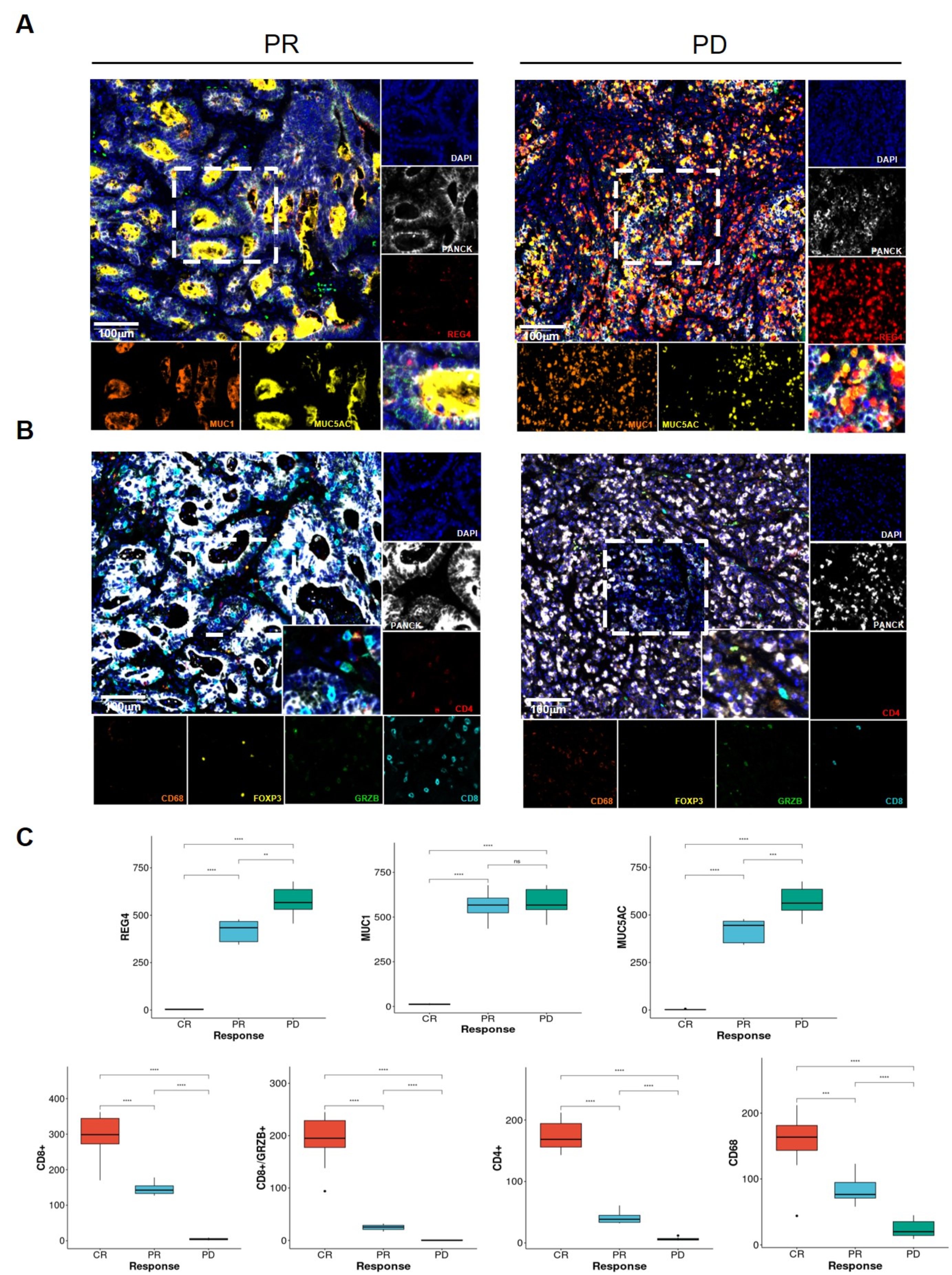

Supplement: Supplementary file 5 — Supplementary Fig. S4 [file 41419_2024_7266_MOESM5_ESM.jpg]

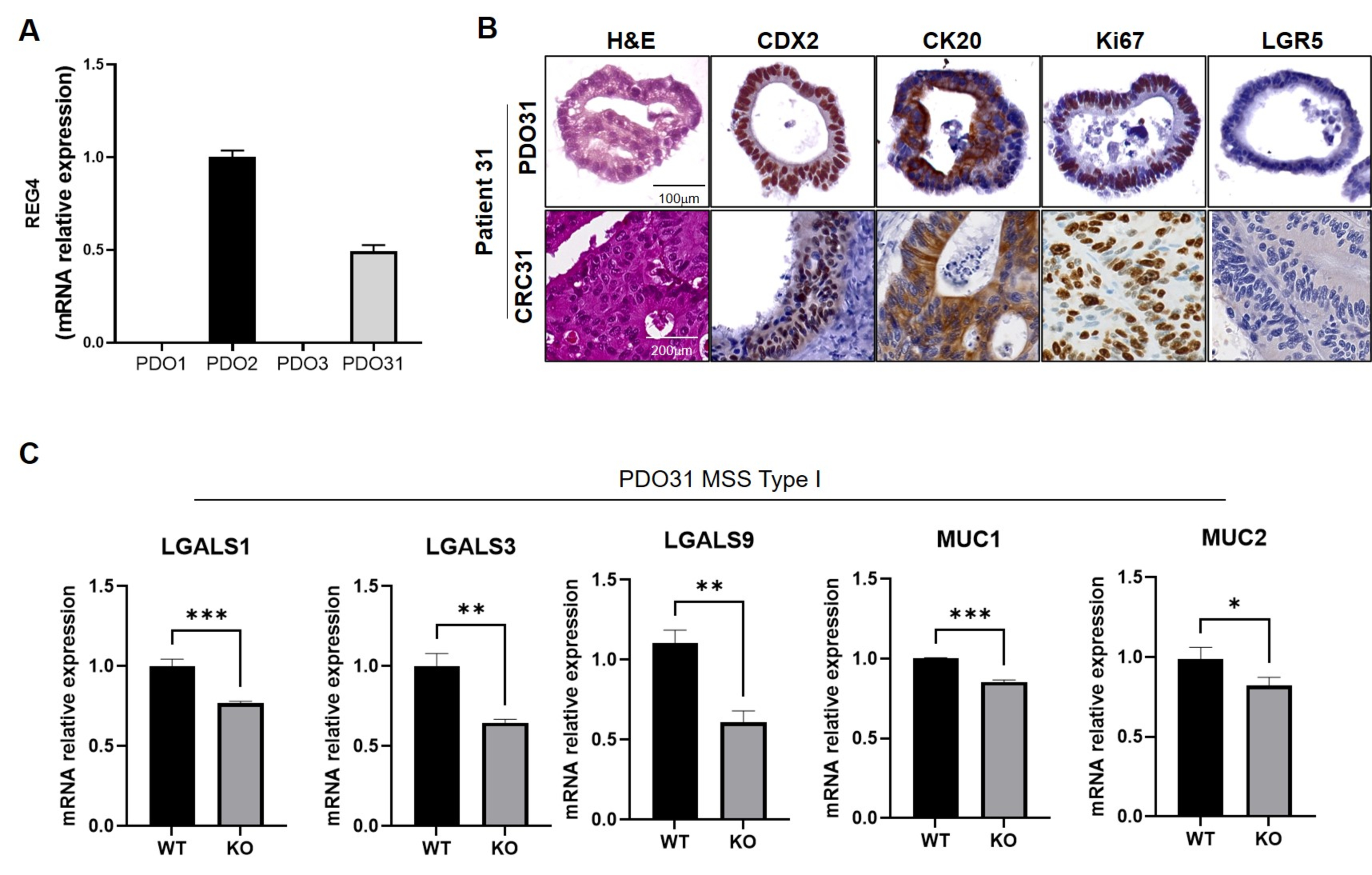

Supplement: Supplementary file 6 — Supplementary Fig. S5 [file 41419_2024_7266_MOESM6_ESM.jpg]
